# Conversion from chronic to episodic migraine in patients treated with erenumab: real-life data from an Italian region

**DOI:** 10.1186/s10194-020-01171-w

**Published:** 2020-08-15

**Authors:** Raffaele Ornello, Alfonsina Casalena, Ilaria Frattale, Valeria Caponnetto, Amleto Gabriele, Giannapia Affaitati, Maria Adele Giamberardino, Maurizio Assetta, Maurizio Maddestra, Fabio Marzoli, Stefano Viola, Davide Cerone, Carmine Marini, Francesca Pistoia, Simona Sacco

**Affiliations:** 1 Department of Applied Clinical Sciences and Biotechnology, University of L’Aquila, Via Vetoio 1, 67100 L’Aquila, Italy; 2 Department of Neurology, ‘G. Mazzini’ Hospital, Teramo, Italy; 3 Neurology Service, ‘SS. Annunziata’ Hospital, Sulmona, Italy; 4 Department of Medicine and Science of Aging, ‘G. D’Annunzio’ University, Chieti, Italy; 5 Department of Neurology, ‘F. Renzetti’ Hospital, Lanciano, Italy; 6 Department of Neurology, ‘S. Pio da Pietrelcina’ Hospital, Vasto, Italy; 7 Department of Neurology, ‘S. Salvatore’ Hospital, L’Aquila, Italy; 8 Department of Medicine, Public Health, Life and Environmental Sciences, University of L’Aquila, L’Aquila, Italy

**Keywords:** Chronic migraine, Calcitonin gene-related peptide, Migraine prevention, Monoclonal antibodies, Erenumab, Real-life study

## Abstract

**Background:**

Most patients treated with erenumab in clinical practice have chronic migraine (CM). We assessed the rate and possible predictors of conversion from CM to episodic migraine (EM) in a real-life study.

**Main body:**

We performed a subgroup analysis of patients treated with erenumab from January 2019 to February 2020 in the Abruzzo region, central Italy. Treatment was provided according to current clinical practice. For the purpose of the present study, we included patients fulfilling the definition of CM for the three months preceding erenumab treatment and with at least 6 months of follow-up after treatment. We assessed the rate of conversion to EM from baseline to Months 4–6 of treatment and during each month of treatment. To test the clinical validity of conversion to EM, we also assessed the decrease in monthly headache days (MHDs), acute medication days, and median headache intensity on a Numerical Rating Scale (NRS). We included in our study 91 patients with CM. At Months 4–6, 62 patients (68.1%) converted from CM to EM; the proportion of converters increased from Month 1 to Month 5. In the overall group of patients, median MHDs decreased from 26.5 (IQR 20–30) to 7.5 (IQR 5–16; *P* < 0.001) compared with baseline, while median acute medication days decreased from 21 (IQR 16–30) to 6 (IQR 3–10; *P* < 0.001) and median NRS scores decreased from 8 (IQR 7–9) to 6 (IQR 4–7; *P* < 0.001). Significant decreases were found both in converters and in non-converters. We found no significant predictors of conversion to EM among the patients’ baseline characteristics.

**Conclusions:**

In our study, two thirds of patients with CM converted to EM during 6 months of treatment with erenumab. MHDs, acute medication use, and headache intensity decreased regardless of conversion from CM to EM.

## Background

Migraine can be classified as episodic (EM) or chronic (CM) according to the number of monthly headache days (MHDs) [1]. CM carries a high burden of disability as it is often associated with medication overuse [2]; besides, patients are often unaware of the availability of effective treatment options [3, 4]. Monoclonal antibodies directed against the calcitonin gene-related peptide (CGRP) or its receptor (CGRPr) are migraine-specific treatments whose efficacy and safety were proven in both EM and CM [5–8]. However, the available real-life studies show that most patients treated in common clinical practice have CM [9–13]. In the present real-life, multicenter study, we assessed the rate and possible predictors of conversion from CM to EM in patients treated with erenumab.

## Methods

### Study population

We performed a subgroup analysis of a real-life study [10]. We included patients aged 18 to 65 years consecutively treated with erenumab in the Headache Centers of Avezzano, L’Aquila, Sulmona, Teramo, Chieti, Lanciano, and Vasto, all located in the Abruzzo region, central Italy, from January 2019 to February 2020. The study was approved by the Internal review Board of the University of L’Aquila with the number 44/2019. All patients signed an informed consent.

Erenumab was provided to patients from the producing company upon reasonable request from the Headache Centers. The drug was provided for patients with migraine with or without aura diagnosed by expert physicians according to the International Classification of Headache Disorders (ICHD-3) criteria [1]. Subjects were selected for treatment if having ≥4 MHDs and ≥ 2 prior preventive treatment failures, according to the European Headache Federation [5] and the American Headache Society [14] criteria.

For the purpose of the present study, we only included patients fulfilling the ICHD-3 criteria for CM at baseline. We excluded from the present study patients with EM and patients with history of CM that had converted to EM in the three months preceding the treatment. The resulting study population was of 91 patients with CM.

### Treatment procedure

Erenumab was administered during in-person visits in a monthly subcutaneous dose of 70 mg, with the option of switching to 140 mg monthly in case of a < 30% decrease in MHDs compared with baseline; patients with several prior preventive treatment failures could start treatment with a 140 mg monthly dose according to the treating physician’s judgement. The treatment was continued at least until Month 6, but we acknowledged the possibility of early withdrawal because of severe adverse events, lack of compliance, or ineffectiveness (< 30% reduction in MHDs and/or lack of satisfaction with treatment). All patients, including those who stopped treatment, were followed-up for 6 months. As this was an observational real-life study, during the period of erenumab treatment the patients were allowed to start, continue, or discontinue concurrent oral preventive treatments for migraine according to clinical indication and also considering patients’ preferences. Patients with medication overuse were not detoxified prior to erenumab treatment, according to current recommendations [5].

### Data collection

For each included patient, we recorded sex, age, migraine and CM duration, migraine frequency and intensity, acute and preventive treatments as reported in the patients’ headache diaries. We assessed attack severity by the 0–10 Numerical Rating Scale (NRS). We also assessed the presence of risk factors for CM, including obesity, sleep disturbances, and depressive symptoms. Obesity was defined as Body Mass Index value ≥30 kg/m^2^; sleep disturbances were defined as any patient report, while depressive symptoms were defined as a Beck Depression Inventory score ≥ 20 or use of antidepressant medications not prescribed for migraine. Data were collected with a clinical interview and then reported on a standardized form with pre-determined answers which was the same for all participating centers. All the recorded data were stored in an anonymized computerized database.

### Statistical analysis

Data from all patients receiving at least one erenumab dose were included in the analyses. Baseline was defined as the monthly mean of the three months preceding erenumab treatment. ‘Converters’ were defined as subjects fulfilling the definition of EM at Months 4–6 of treatment, while the remaining patients, including those discontinuing erenumab treatment, were defined as ‘non-converters’. Calculations at Months 4–6 were based on the mean values of the three months. The primary outcome of the study was the overall rate of converters at Months 4–6; the secondary outcomes of the study were to evaluate the rate of converters per each month of treatment from Month 1 to Month 6 and the rates of high-frequency (HFEM; 8–14 MHDs), medium-frequency (MFEM; 4–7 MHDs), and low-frequency EM (LFEM; 0–3 MHDs) at Months 4–6 and per each month of treatment. To verify the clinical validity of assessing CM conversion, we compared the treatment outcomes (decrease in MHDs, days of acute medication, and median NRS from baseline to Months 4–6) in converters and in non-converters. Assessment timepoints were chosen in accordance to the STRIVE trial [15].

Categorical data were reported as numbers and proportions, while continuous data were reported as medians and interquartile ranges (IQRs). We used the chi-square test to compare categorical variables and the Mann-Whitney U test to compare medians. Statistical significance was set at *P* < 0.05. We estimated that a sample size of 43 subjects would be adequate to detect a 68% conversion rate, as previously reported [16], with a 95% confidence interval and 90% precision.

## Results

All the 91 patients with CM were followed-up for at least six months in the absence of losses to follow-up; 11 patients (12.1%) discontinued the treatment before Month 6 due to ineffectiveness (10 patients) or adverse events (1 patient). Table 1 reports the baseline characteristics of the study patients.

**Table 1:** Characteristics of the study patients

Characteristics (total patients = 91)	
Female, *n* (%)	80 (87.9)
Age, median (IQR)	49 (39–54)
Years of migraine history, median (IQR)	28.5 (20–34)
Years of CM history, median (IQR)	10 (4–19)
Baseline MHDs, median (IQR)	26.5 (20–30)
Baseline acute medication days, median (IQR)	21 (16–30)
Baseline NRS, median (IQR)	8 (7–9)
Aura, *n* (%)	28 (30.8)
Allodynia, *n* (%)	35 (38.5)
Medication overuse, *n* (%)	71 (78.0)
Previous preventive treatment failures, *n* (%)	
2	31 (43.1)
3	24 (26.4)
4	28 (30.8)
> 4	8 (8.8)
Botulinum toxin failure, *n* (%)	39 (42.9)
Concurrent oral preventive treatments at baseline, *n* (%)	30 (33.0)
Obesity, *n* (%)	13 (14.3)
Sleep disturbances, *n* (%)	33 (36.3)
Depressive symptoms, *n* (%)	19 (20.9)

*CM* indicates chronic migraine, *IQR* interquartile range, *MHD* monthly headache days, *NRS* Numerical Rating Scale

**Fig. 1:**
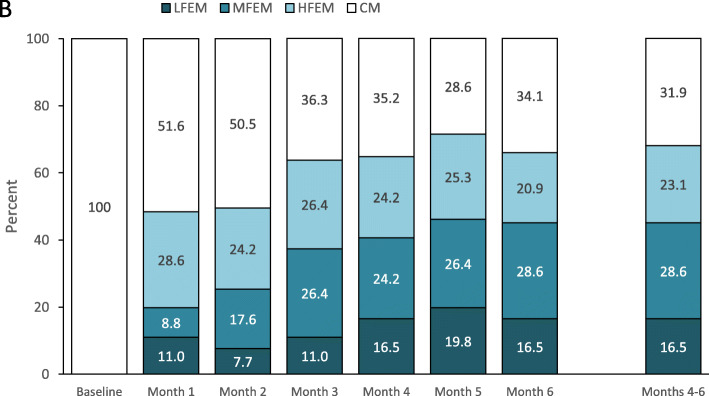
Rates of conversion to episodic migraine at Months 4–6 and after each month of treatment according to monthly headache days. HFEM indicates high-frequency episodic migraine (8–14 monthly headache days); LFEM, low-frequency episodic migraine (0–3 monthly headache days); MFEM, medium-frequency episodic migraine (4–7 monthly headache days)

Sixty-two (68.1%) patients were converters at Months 4–6. Monthly converters increased from 44 (48.4%) at Month 1 to 65 (71.4%) at Month 5 (Fig. 1). At Months 4–6, 15 (16.5%) patients achieved the status of LFEM, 26 (28.6%) MFEM, and 21 (23.1%) HFEM. Figure 1 shows the proportion of patients with LFEM, MFEM, and HFEM after each month of treatment. Thirty-eight (41.8%) patients reached the converter status without needing erenumab dose increase from 70 mg to 140 mg monthly, while 24 (26.4%) patients needed a dose increase; all non-converters increased the erenumab dose during follow-up. Concurrent migraine preventive treatments were discontinued in 11 (12.1%) patients.

**Fig. 2:**
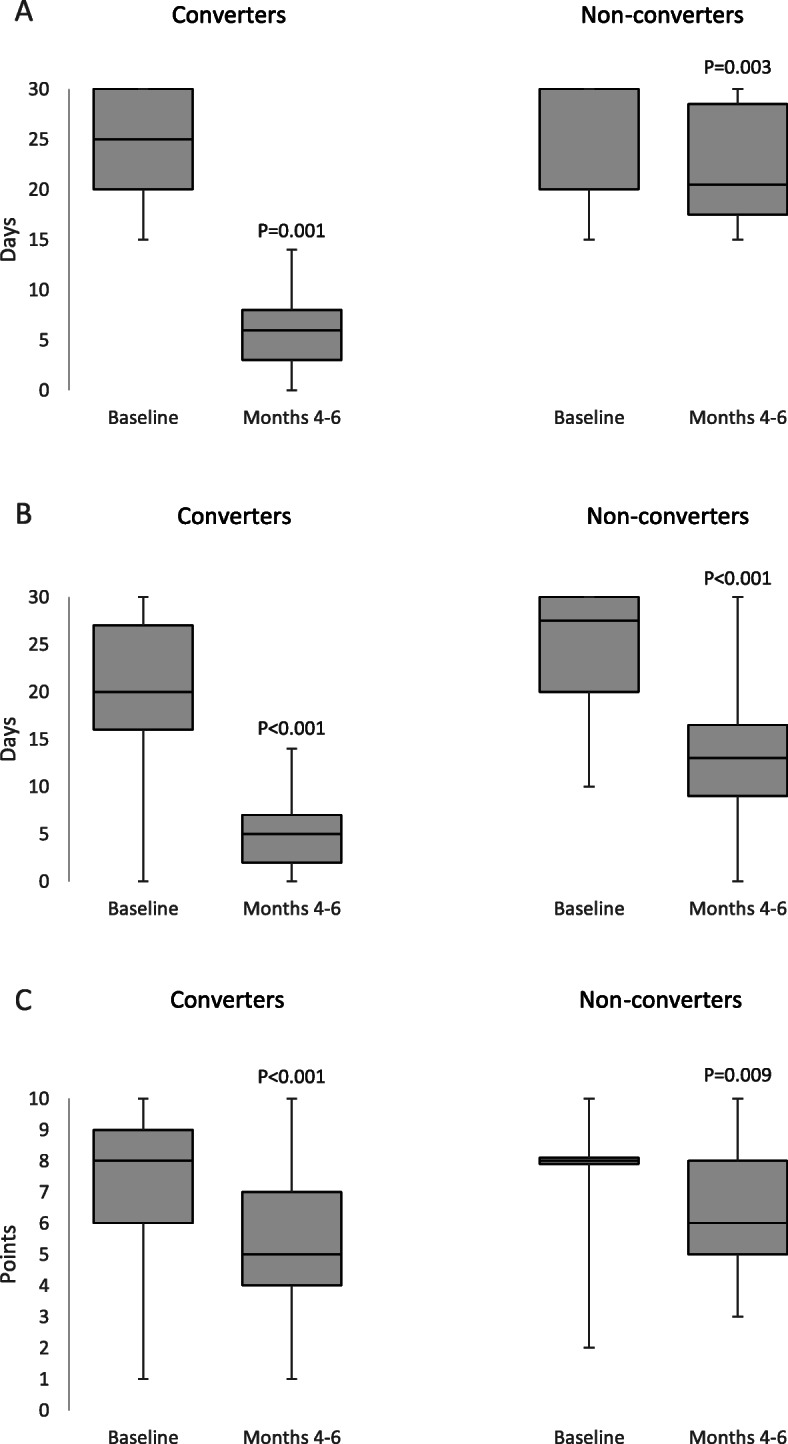
Decrease in median headache days **a**, acute medication days **b**, and headache intensity on a Numerical Rating Scale **c** according to converter status in the present study

At Months 4–6, median MHDs decreased from 26.5 (IQR 20–30) to 7.5 (IQR 5–16; *P* < 0.001) compared with baseline in the overall group, from 25 (IQR 20–30) to 6 (IQR 3–8; *P* < 0.001) in converters, and from 30 (IQR 20–30) to 20.5 (IQR 17.5–28.5; *P* = 0.003) in non-converters (Fig. 2-a). Median acute medication days decreased from 21 (IQR 16–30) to 6 (IQR 3–10; *P* < 0.001) in the overall group, from 20 (IQR 16–27) to 5 (IQR 2–7; *P* < 0.001) in converters, and from 27.5 (IQR 20–30) to 13 (IQR 9–16.5; *P* < 0.001) in non-converters (Fig. 2b). In detail, median days of triptan use changed from 4 (IQR 0–20) to 2 (IQR 0–6; *P* < 0.001) in the overall group, from 6 (IQR 0–20) to 1 (IQR 0–5; *P* < 0.001) in converters, and from 0 (IQR 0–15) to 3 (IQR 0–8; *P* = 0.043) in non-converters, while median days of common analgesic use decreased from 11 (IQR 4–25) to 3 (IQR 1–6; *P* < 0.001) in the overall group, from 8 (IQR 0–20) to 1 (IQR 0–4; *P* < 0.001) in converters, and from 20 (IQR 10–30) to 11 (IQR 6–18; *P* = 0.002) in non-converters. Median NRS decreased from 8 (IQR 7–9) to 6 (IQR 4–7; *P* < 0.001) in the overall group, from 8 (IQR 6–9) to 5 (IQR 4–7; *P* < 0.001) in converters, and from 8 (IQR 8–8) to 6 (IQR 5–8; *P* = 0.009) in non-converters (Fig. 2c). All the 46 converters and 16 (64.0%) of the 25 non-converters with medication overuse withdrew from that condition.

Univariate comparisons showed no differences in baseline characteristics between converters and non-converters (Table 2).

**Table 2:** Baseline characteristics of converters versus non-converters to episodic migraine during the treatment

Characteristic	Converters (*n* = 62)	Non-converters (*n* = 29)	*P* value
Female, *n* (%)	54 (87.1)	26 (89.7)	0.727
Age, median (IQR)	47 (38–51)	53 (42–57)	0.060
Years of migraine history, median (IQR)	28 (20–33)	29 (20–37)	0.435
Years of CM history, median (IQR)	8 (5–12)	15 (4–22)	0.099
MHDs, median (IQR)	25 (20–30)	30 (20–30)	0.360
Acute medication days, median (IQR)	20 (16–27)	27.5 (20–30)	0.063
Baseline NRS, median (IQR)	8 (6–9)	8 (8–8)	0.349
Aura, *n* (%)	18 (29.0)	10 (34.4)	0.600
Allodynia, *n* (%)	24 (38.7)	11 (37.9)	0.943
Medication overuse, *n* (%)	46 (74.2)	25 (86.2)	0.197
Prior preventive treatment failures, *n* (%)			0.954
2	21 (33.9)	10 (34.5)	
> 2	41 (66.1)	19 (65.5)	
Botulinum toxin failure, *n* (%)	26 (41.9)	13 (44.8)	0.795
Obesity, *n* (%)	9 (14.5)	4 (13.8)	0.999
Sleep disturbances, *n* (%)	19 (30.6)	14 (48.3)	0.103
Depressive symptoms, *n* (%)	13 (21.0)	6 (20.7)	0.976

*CM* indicates chronic migraine, *IQR* interquartile range, *MHD* monthly headache days, *NRS* Numerical Rating Scale

## Discussion

Our data show that two thirds of patients with CM convert to EM during a 6-month treatment with erenumab. The proportion of patients converting to EM was about half at Month 1 and increased up to three quarters at Month 5. All converters withhold medication overuse. The high rate of conversion to EM in our population of difficult-to-treat patients with a long history of CM and multiple prior preventive treatment failures, including botulinum toxin in ≥40% of cases, supports the efficacy of erenumab for the preventive treatment of patients with CM, as shown in randomized controlled trials [17–22] and real-life studies [10–13]. We also found that at Months 4–6 16.5% of patients achieved a status of LFEM, while 28.6% achieved a status of MFEM, which indicates a high treatment benefit and a substantial improvement in the patients’ quality of life.

Notably, the treatment decreased headache frequency, intensity, and use of triptans and common analgesics in both converters and non-converters, suggesting that even patients who do not convert to EM may have benefits from erenumab treatment. Erenumab treatment also had a relevant effect on medication overuse withdrawal both in converters and in non-converters. With regard to those findings, it should be noted that CM and EM are not distinct entities, as suggested by the frequent fluctuations between the two conditions [23] and the similar levels of disability associated with CM and HFEM [24].

We found no predictors of conversion to EM, even when considering characteristics associated with CM such as sleep disturbances, obesity, and depressive symptoms [25]. Further larger studies are needed to assess reliable predictors of favorable response to anti-CGRP treatment in order to maximize treatment outcomes.

We collected a sample of patients treated according to clinical practice in a multicenter study. Besides, to ensure reliable statistical estimates, we chose to perform nonparametric tests such as the Wilcoxon test. However, our study also suffers from several limitations. Due to low numbers, we could not assess the role of concurrent migraine preventive treatments and of several factors influencing migraine, such as seasonality or life events. We also could not assess the added contribution of escalating erenumab dose from 70 mg to 140 mg monthly, which might have been relevant in patients with multiple prior preventive treatment failures [26]. Besides, we did not have data on patient-reported outcomes, such as quality of life measures, which would have been useful to better quantify the benefit of erenumab. Better measures are needed to exactly quantify the response to anti-CGRP migraine preventive treatments and to identify factors that can predict good treatment outcomes. Lastly, we cannot exclude that in some patients the conversion to EM was due to the natural fluctuation of the disease or to placebo effect rather than to the treatment itself.

## Conclusion

In our study, two thirds of patients with CM converted to EM during 6 months of treatment with erenumab. Patients reported a decrease in MHDs, headache intensity, and acute medication use irrespective of their converter status.

## Data Availability

Anonymized data operated or analyzed during this study are available from the Authors upon reasonable request.

## Abbreviations

CGRPr: Calcitonin gene-related peptide receptor
CM: Chronic migraine
EM: Episodic migraine
HFEM: High-frequency episodic migraine
ICHD-3: International Classification of Headache Disorders, 3rd Edition
LFEM: Low-frequency episodic migraine
MFEM: Medium-frequency episodic migraine
MHDs: Monthly headache days
NRS: Numerical Rating Scale
